# Changes in Postharvest Quality and Physiological Attributes of Strawberry Fruits Influenced by l‐Phenylalanine

**DOI:** 10.1002/fsn3.4564

**Published:** 2024-11-04

**Authors:** Karim Manda‐Hakki, Hamid Hassanpour

**Affiliations:** ^1^ Department of Horticultural Sciences, Faculty of Agriculture Urmia University Urmia Iran

**Keywords:** antioxidant, ascorbate peroxidase, l‐phenylalanine, malondialdehyde, shelf life, storage

## Abstract

Strawberry (*Fragaria × ananassa*) is a popular fruit with rich nutrients and a delicious taste. But this fruit is very vulnerable to diseases and decay. Therefore, l‐phenylalanine (Phe) (0, 4, 8 mM) was considered to improve biochemical characteristics and activity of antioxidant enzymes in strawberry fruit cv. Sabrina under cold storage (5, 10, 15 days). After treatment and storage, traits including weight loss, total phenol (TP), antioxidant capacity, ascorbic acid, total anthocyanin (TA), total flavonoid (TF), malondialdehyde (MDA), soluble protein content and antioxidant enzymes such as superoxide dismutase (SOD), catalase (CAT), ascorbate peroxidase (APX), and guaiacol peroxidase (POD) and phenylalanine ammonialyase (PAL) were evaluated at 5‐day intervals. Our findings showed that the treatment of l‐phenylalanine in different concentrations prevented the weight loss of the fruit compared to the control and maintained and increased TP, antioxidant capacity, ascorbic acid, TA, TF, soluble protein and SOD, CAT, APX, POD, and PAL enzymes activity. Also, Phe decreased the MDA content and peroxidation of lipid. The results showed that 4 mM Phe is the best treatment for improving phytochemical characteristics and maintaining fruit quality. The findings indicated that Phe treatment may be useful to improve quality and increase postharvest shelf life in strawberry fruits.

## Graphical abstract

This study showed that the exogenous application of l‐phenylalanine increased the content of total phenol, total flavonoid, total anthocyanin, antioxidant capacity, ascorbic acid, and soluble protein, and reduced MDA levels. It prevented fruit weight loss and increased the activity of antioxidant enzymes (CAT, APX, POD, and SOD) and PAL.

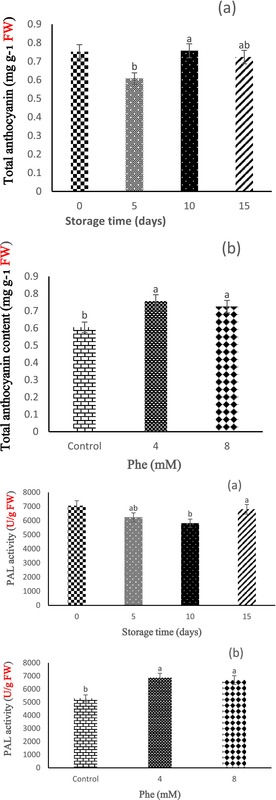

## 1 Introduction

Strawberry (*Fragaria × ananassa*) belongs to the Rosaceae family and is a popular fruit with rich nutrients and a delicious taste (Chang et al. 2023). The high content of compounds such as phenol, flavonoids, vitamins, and anthocyanin in strawberries has made it one of the most important fruits in the world market. However, their shelf life is low due to their nature, which may be one of the most important reasons, the high respiration rate of fruits. Other important factors that lead to changes in the biochemical characteristics of strawberries after harvest include physical damage and infections induced by diseases and pests (Piechowiak et al. 2019; Chu et al. 2020).

Food waste is one of the most important global challenges. Annually, on average, more than 9 million tons of fruits become waste after harvest (Bishop, Styles, and Lens 2021). Therefore, to increase the shelf life of fruits, and maintain their nutritional value, aroma and flavor, and their biochemical properties, it is clear that new postharvest technologies are needed (Brizzolara et al. 2020; Holler et al. 2023).

After harvesting strawberries and leaving them at room temperature, the fruits gradually lose water and become softer, and as a result, the rotting of the fruits is increased (Gol et al. 2013). However, exposure to low temperatures slightly slows down this process but it is not very significant. Therefore, treating fruits or using postharvest technologies along with storage at a low temperature can have considerable effects and increase shelf life. Chemical compounds are the best postharvest technologies applied to various products (Kahramanoglu 2019).

Among the chemical compounds used to increase storage after harvesting fruits are amino acids. Since previous years, farmers have used amino acids and related products, as organic and nitrogenous compounds to improve the growth, development, and performance of various crops. These compounds play a significant role in the availability of some elements and minerals (Cerdna et al. 2009). In addition to protein biosynthesis, amino acids contribute to plant hormone biosynthesis as intermediary molecules (Taiz et al. 2017).

Amino acids are building blocks of proteins that play a vital role in creating resistance to diseases and pests, increasing antioxidant capacity and phenolic compounds. Phenylacetic acid is the first intermediate compound for the synthesis of phenolic compounds, flavonoids, anthocyanin, lignin, and tannin, and the amino acid phenylalanine (Phe) is the main constituent of this biologically important molecule (Garde‐Cerdan et al. 2014). Also, the aromatic amino acid Phe plays a vital role in the biosynthesis of phenolic compounds derived from the phenylpropanoid pathway. The first enzyme that converts this amino acid into phenolic compounds such as phenols, flavonoids, and anthocyanins is phenylalanine ammonialyase (PAL) which is induced by various biotic and abiotic stresses (Aghdam et al. 2019). Also, Phe is known as one of the most important amino acids in the biosynthesis of aromatic compounds and antioxidants and plays an important role in defense systems (Aghaei et al. 2019).

A previous study conducted by Sogvar et al. (2020) determined that postharvest application of Phe on plums improved the firmness and quality of fruits and 7.5 mM treatment reduced frost injury. Also, the results showed that total flavonoid (TF), phenol, anthocyanin, ascorbic acid, proline content, PAL, and CAT enzyme activities of treated fruits with Phe were higher than untreated fruits. Furthermore, studies conducted on different fruits such as avocado, citrus, and mango have determined that Phe treatment decreases fungal decay and increases their resistance to pests and diseases (Patel et al. 2020). Postharvest treatment of avocados with Phe led to an increase in the defense system against pathogens and increased the shelf life of avocado fruits at sub‐optimal temperatures (Saidi et al. 2021). Ahmadkhani et al. (2023) reported that Phe treatment increased the content of total anthocyanin (TA), flavonoid, phenol, and total soluble solids of blueberry fruits and prevented the reduction of ascorbic acid content. Also, Phe preserved the membrane integrity by reducing hydrogen peroxide content and frost injury and increasing the proline content, which had a direct effect on reducing the weight loss of fruits and increasing the storage life of fruits. Previously, in eggplant (*Solanum melongena* L.) fruits, it was found that the treatment of 7.5 mM Phe is suitable for maintaining fruit quality, delaying weight loss and frost damage, and increasing storage life (Najafi et al. 2021).

Based on the review of the literature, it was found that few studies have been done on the effect of Phe on strawberry fruit during cold storage. Therefore, in this research, we are looking for an appropriate concentration of Phe treatment to increase and maintain the quality and reduce postharvest waste of strawberry fruits. Hence, we aim to reduce costs by replacing cheap and healthy methods with postharvest technology.

## 2 Materials and Methods

### 2.1 Plant Materials and Treatment

**FIGURE 1:**
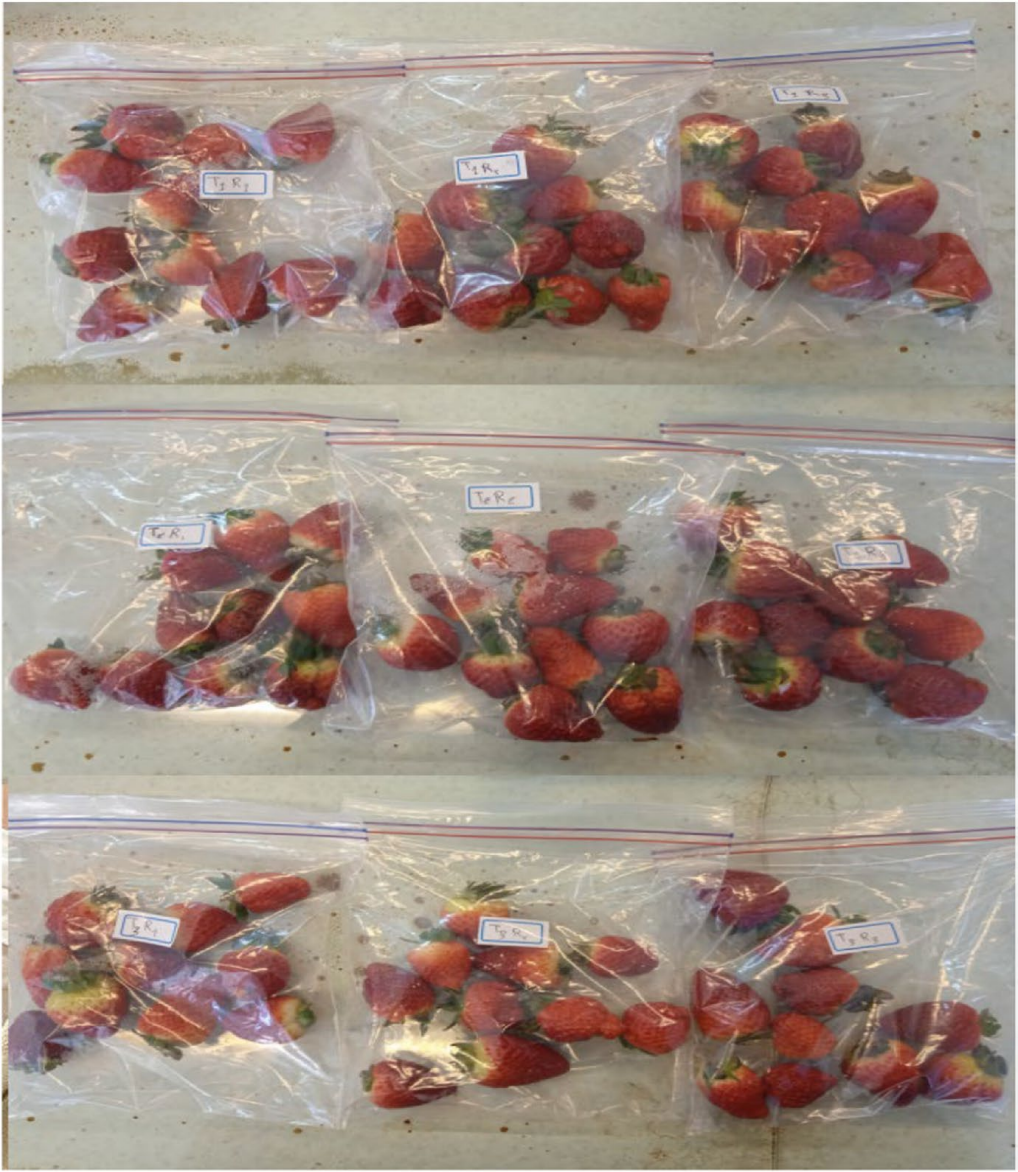
Postharvest photographs of Sabrina strawberry fruits in control (T1) and L‐phenylalanine treated group (T2, 4 mM; T3, 8 mM) during cold storage.

Strawberry (*Fragaria × ananassa* cv. Sabrina) fruits were harvested from a commercial greenhouse in Urmia, Iran at physiological maturity (Figure 1). The fruits were transported to the lab following the necessary precautions to prevent mechanical damage. Undamaged fruits with uniform size were separated and divided into three groups. The first group was treated as a control and the other two groups were treated with l‐phenylalanine (4 and 8 mM). After drying for 2 h, the treated fruits were placed in zipped bags and then stored for 15 days at a temperature of 4°C and a relative humidity of 90%–95% in cold storage. Finally, measurable parameters were determined after 5, 10, and 15 days of storage. Also, three biological replicates were used for each treatment.

### 2.2 Weight Loss

The weight loss rate was measured by the following formula: weight loss percentage = (M1 − M2/M1) × 100, where M1 represents the weight of the sample at 0 days and M2 represents the weight of the final fruits (Meng et al. 2007).

### 2.3 Total Phenol, Total Flavonoids, Total Anthocyanin Content, and Antioxidant Activity

According to the Folin Ciocalteu method, 50 μL of extracts was added to 4.2 mL distilled water, and then 10% (v/v) diluted Folin reagent (250 μL) and vortexed for 10 s to mix. Then 20% Na_2_CO_3_ (500 μL) was added and mixed well. The mixtures were reacted for 30 min at room temperature in the dark. The mixture was read by spectrophotometer (Model Pharmacia LKB. Novaspec II) at 765 nm against a blank. Gallic acid (GA) was the standard compound for the calculation of phenols. TP content was expressed as mg GAE/100 g FW (Dzah et al. 2023).

Shin, Ghimeray, and Park (2014) method was used to determine TF with minor modifications. First, 500 μL of the extract was added to 150 μL of 5% sodium nitrite, and in the next step, after 5 min, 300 μL of 10% aluminum chloride was added to it. Then, after 5 min, 1 mL of 1 M NaOH was added, and finally the final volume of the solution reached 5 mL with distilled water. The absorbance of the samples was measured by spectrophotometer (model Pharmacia LKB. Novaspec II) at 510 nm. TF content was expressed as mg catechin per 100 g FW.

The pH difference method was used to determine TA content. In this method, two buffers with pH 1 and 4.5 were first prepared, in the next step, 100 μL of the supernatant solution was added to 2.5 mL of buffer with pH = 1, and the absorbance was read at two wavelengths of 530 and 700 nm by spectrophotometer (Model Pharmacia LKB. Novaspec II), and then the same procedure was repeated for pH = 4.5. TA content was expressed as mg of cyanide 3‐glucoside per gram using the following formula (Giusti and Wrolstad 2001):

$$\mathrm{TA}=\left(A\times \mathrm{MW}\times \mathrm{DF}\times 100\right)/\left(\varepsilon \times 100\right)$$

where *A* = absorption, MW = molecular weight of cyanidin 3‐glucoside (449.2), DF = dilution factor, *ε* = molar extinction coefficient of cyanidin 3‐glucoside (29,600).

The iron‐reducing antioxidant property (FRAP) method was used to determine antioxidant activity. In general, acetate buffer (300 mM), solution of 2,4,6‐tri(2‐pyridyl)s‐triazine (TPTZ) (10 mM) in HCl (40 mM), and a freshly prepared FeCl_3_ × 6 H_2_O solution (10 mM) were mixed with a ratio of 10:1:1. Sample extracts were diluted (1:10, sample: acetate buffer); in the next step, 50 μL of each sample was mixed with 3.5 mL of FRAP solution for 10 min at 37°C to react. The absorbance of the iron TPTZ complex was read by spectrophotometer (Model Pharmacia LKB. Novaspec II) at 593 nm against blanks. The amount of antioxidant activity was expressed as mM iron per 100 g FW (Dzah et al. 2020).

### 2.4 Ascorbic Acid Content

The content of ascorbic acid was measured by the spectrophotometric (Model Pharmacia LKB. Novaspec II) method. Briefly, 100 μL of fruit extract was mixed with 10 mL of 1% metaphosphoric acid, 1 mL of the resulting solution was mixed with 9 mL of 50 μM 2, 6‐dichloroindophenol and vortexed for a few seconds, and then the absorbance was read at 515 and was expressed as mg of ascorbic acid per g (Klein and Perry 2006).

### 2.5 Malondialdehyde Content

The thiobarbituric acid (TBA) method was used to measure malondialdehyde (MDA) content. MDA content was expressed as nmol/g FW (Hu et al. 2021).

### 2.6 Enzyme Activities Assay and Soluble Protein Content

To measure the soluble protein content and enzyme activity, enzyme extracts were used, which were prepared in 2 mL of prepared potassium phosphate buffer (50 mM, pH = 7.5) containing 1% polyvinyl pyrrolidone (PVP), 50 mM Tris, and 1 mM EDTA.

#### 2.6.1 Soluble Protein Content

The method of Bradford (1976) was used to measure the soluble protein in the extracts. After extraction, 50 μL of the extract was first added to 2950 μL of Bradford solution and mixed thoroughly, and after 10 min, the absorbance of the mixture was read by spectrophotometer (Model Pharmacia LKB. Novaspec II) at 595 nm. To determine the protein content (mg/g FW), bovine serum albumin (BSA) was used as a standard.

#### 2.6.2 Superoxide Dismutase Activity

SOD activity was measured by the method of Beauchamp and Fridovich (1971) (EC 1.15.1.1). Based on this method, 3 mL of reaction mixture containing 13 mM methionine, 2 μM riboflavin, 50 mM phosphate buffer (pH 7.5), 0.1 μM EDTA, and 75 μM nitro blue tetrazolium (NBT) were prepared to inhibit the ability of the enzyme extract. The photochemical reduction of NBT was determined by measuring the absorbance at 560 nm. Finally, SOD activity was measured by subtracting the reducing NBT in the presence of light and without the presence of enzyme extract from the reduction of NBT with enzyme extract (*E* = 2.8 mM/cm).

#### 2.6.3 Catalase Activity

CAT enzyme activity (EC 1.11.1.6) was measured by H_2_O_2_ decomposition at 240 nm for 60 s. A reaction mixture (3 mL) was prepared with 100 μL of H_2_O_2_ (300 mM) and 100 μL of enzyme extract by adding 2.79 mL of 50 mM phosphate buffer (pH 7.0, containing 2 mM EDTA). CAT activity was determined using *E* = 39.4 mM/cm as the extinction coefficient (Aebi 1984).

#### 2.6.4 Ascorbate Peroxidase Activity

To measure the activity of ascorbate peroxidase (APX) (EC 1.11.1.11), the method of Nakano and Asada (1981) was used, based on H_2_O_2_‐dependent ascorbate oxidation at 290 nm. Briefly, the reaction solution containing 100 μL ascorbate (7.5 mM), 2.6 mL 25 mM phosphate buffer (pH 7.0), 100 μL H_2_O_2_ (300 mM), 2 mM EDTA, and 100 μL enzyme extract was prepared. APX activity was determined based on the extinction coefficient (2.8 mM/L/cm).

#### 2.6.5 Guaiacol Peroxidase Activity

The method of Nakano and Asada (1981) was used to measure POD activity with a slight modification. For this purpose, a reaction solution containing 3 μL of 50 mM phosphate buffer (pH = 7) with 50 μL of pure guaiacol and 50 μL of 3 μM hydrogen peroxide was prepared and mixed in an ice bath, and then, 50 μL of enzyme extract was added to it. POD activity was recorded spectrophotometrically (Model Pharmacia LKB. Novaspec II) as an increase in absorbance within 1 min at 420 nm. One POD enzyme unit equals the degradation of 1 μM tetragonal in 1 min.

#### 2.6.6 Phenylalanine Ammonialyase Activity

The method of Cunha et al. (1996) was used to determine PAL activity. First, 1 mL of 50 mM potassium phosphate buffer (pH = 7), 0.5 mL of 10 mM Phe, 0.4 mL of double distilled water, and 100 μL of enzyme extract were prepared and mixed, and then kept for 1 h in a 37°C bath. In the next step, the reaction was stopped by adding 0.5 mL of 6 M hydrochloric acid. Finally, the absorbance of the samples was measured at 260 nm, and the PAL activity was expressed as nmol/g/min FW.

### 2.7 Statistical Analysis

Data obtained from the experiment were analyzed using SAS (version 9.4), and Duncan's multiple range test at 5% was used to compare means. Also, Statgraphics (19 X64) software was used to evaluate the principal component of the data, and SPSS (Statics 27) software was used to measure the correlation.

## 3 Results

### 3.1 Weight Loss

**FIGURE 2:**
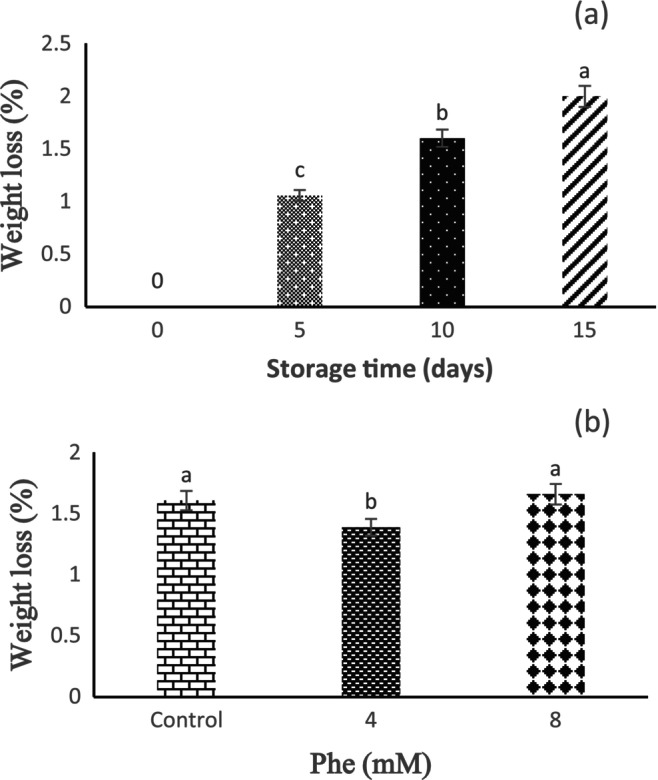
The effect of storage time (a) and L‐phenylalanine (Phe) treatment (b) on weight loss of Sabrina strawberry fruits.

Based on the results, the effect of storage time on fruit weight loss was significant at 1% level. With the passage of storage time, the percentage of fruit weight loss also increased, so the lowest weight loss was observed in 5 days of storage and the highest weight loss was observed at the end of storage (15 days) (Figure 2a). The effect of Phe treatment on fruit weight loss was significant at the level of 5%, and it was observed that Phe treatment reduces the percentage of fruit weight loss. Also, the results revealed that the highest and lowest weight losses were observed in the 8 and 4 mM Phe treatments, respectively, compared to the control (Figure 2b).

### 3.2 Total Phenol, Total Flavonoids, Total Anthocyanin Content, and Antioxidant Activity

**FIGURE 3:**
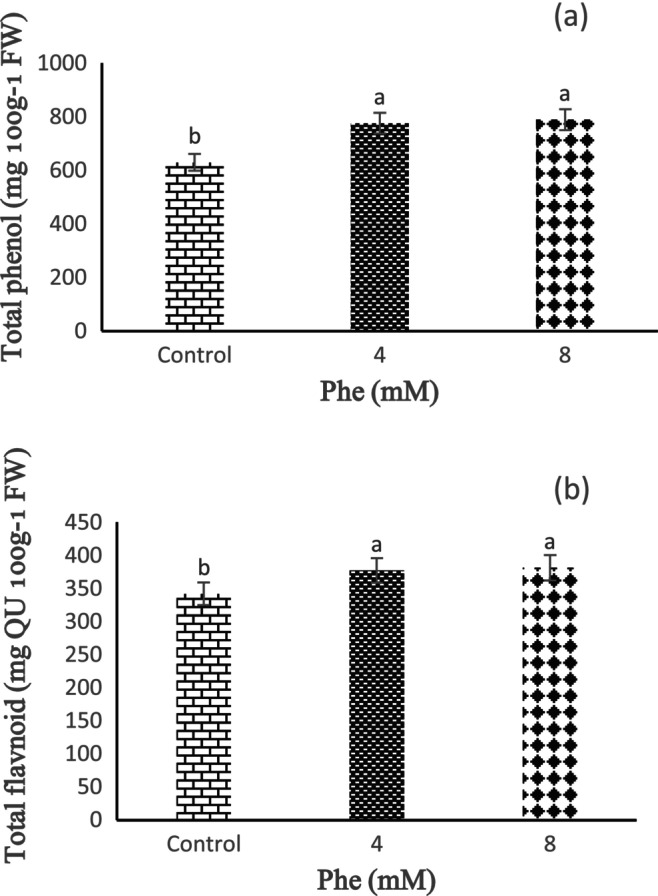
The effect of L‐phenylalanine (Phe) on total phenol content (a) and total flavonoid (b) of Sabrina strawberry fruits.

Based on our findings, storage time has no statistically significant effect on TP content. Meanwhile, the effect of Phe treatment on TP content was statistically significant (*p* < 0.01). The results indicated that Phe treatment maintains or increases TP content. Based on these results, the highest and lowest contents of TP were determined in 8 and 4 mM Phe treatments, respectively, compared to the untreated fruits (Figure 3a).

The results indicated that the storage time did not have a statistically significant effect on the TF content. Meanwhile, the Phe treatment effect on TF content was statistically significant (*p* < 0.01). According to these findings, with the increase in Phe concentration, the content of TF increases compared to the control, so the highest content of TF was observed in the treatment of 8 mM Phe and the lowest content was revealed in 4 mM Phe (Figure 3b).

**FIGURE 4:**
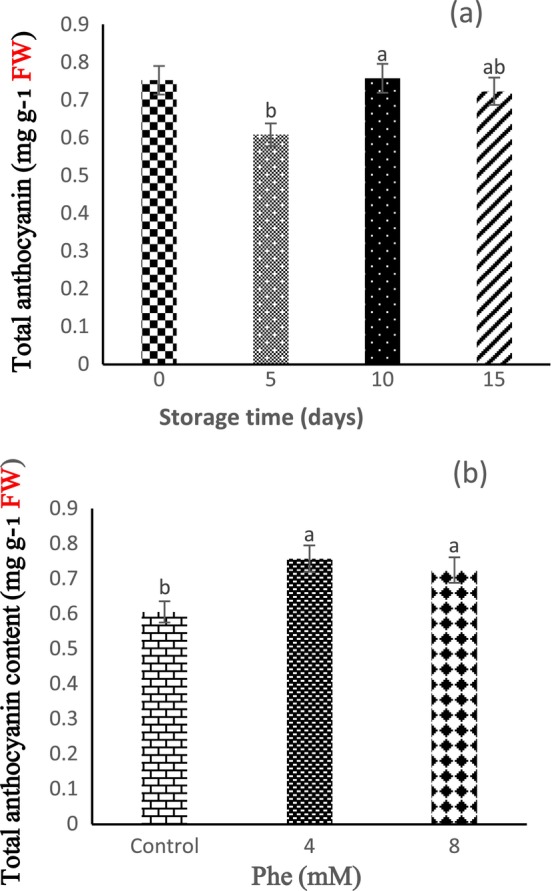
The effect of storage time (a) and L‐phenylalanine (Phe) treatment (b) on total anthocyanin content of Sabrina strawberry fruits.

According to the obtained results, the storage time did not have a statistically significant effect on the content of TA (*p* < 0.05). These results revealed that the lowest TA content was determined in 5 days of storage, and the highest content of TA was seen in 10 days of storage (Figure 4a). It was also observed that Phe treatment increases the content of TA, and based on our findings, 4 and 8 mM Phe treatments revealed the highest and lowest TA contents, respectively (Figure 4b).

**FIGURE 5:**
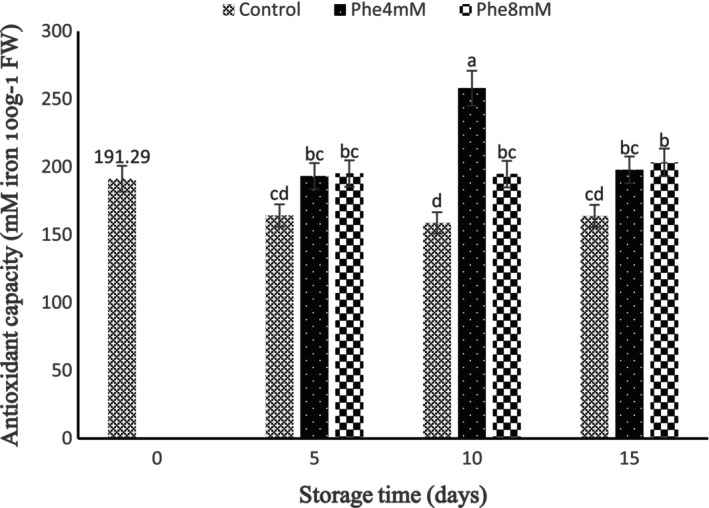
Interaction effect of storage time and L‐phenylalanine (Phe) treatment on antioxidant capacity.

Based on the results, the interaction effect of storage time and Phe treatment on antioxidant activity was statistically significant (*p* < 0.01). The treatment of 4 mM Phe in 10 days of storage revealed the highest antioxidant activity, while the lowest antioxidant activity was revealed in 4 mM Phe treatment during 5 days of storage. Our findings revealed that Phe treatments increased the activity of antioxidants in 5 days of storage compared to the control, although the effect of the 8 mM treatment was greater. An increasing trend was determined in 10 days of storage, and the 4 mM Phe was more effective than the 8 mM Phe treatment, while in 15 days of storage, this trend decreased and the 8 mM Phe treatment had a better effect than the 4 mM Phe treatment (Figure 5).

### 3.3 Ascorbic Acid Content

**FIGURE 6:**
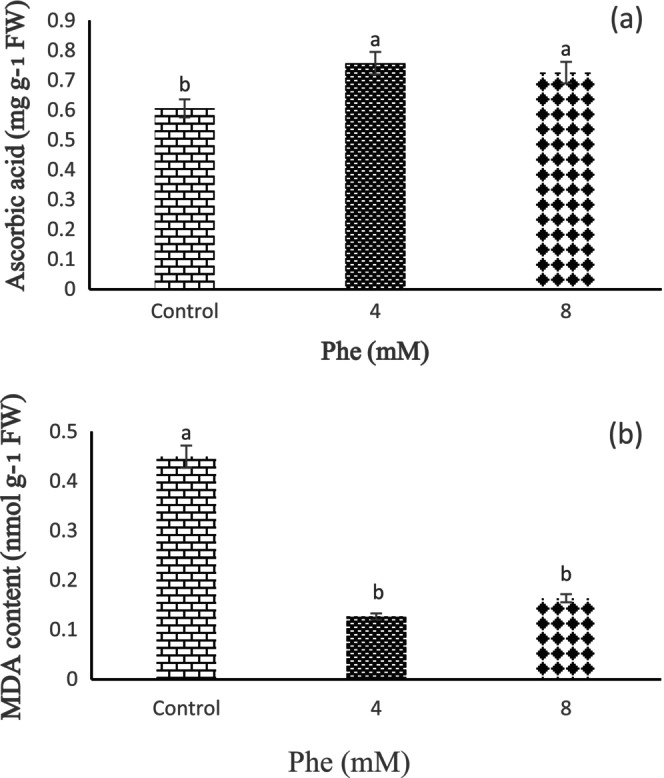
The effect of L‐phenylalanine (Phe) on ascorbic acid (a) and MDA content (b) of Sabrina strawberry fruits.

According to the obtained results, the storage time effect on ascorbic acid was not statistically significant, meanwhile, Phe treatments had a statistically significant effect on ascorbic acid content (*p* < 0.01). The fruits treated with Phe maintained more ascorbic acid than the control. The results showed that the fruits treated with 4 mM Phe had the highest ascorbic acid content. Also, the lowest ascorbic acid content was seen in the 8 mM Phe treatment (Figure 6a).

### 3.4 MDA and Soluble Protein Content

According to the obtained results, the Phe treatment effect on MDA content was statistically significant at 1%. The results showed that Phe treatment has a positive effect in reducing the level of MDA and lipid peroxidation, so all Phe treatments decreased the level of MDA and lipid peroxidation. According to these results, 8 mM Phe treatment had the lowest and 4 mM Phe treatment had the highest effect on MDA content in strawberry fruits compared to the control during storage (Figure 6b).

**FIGURE 7:**
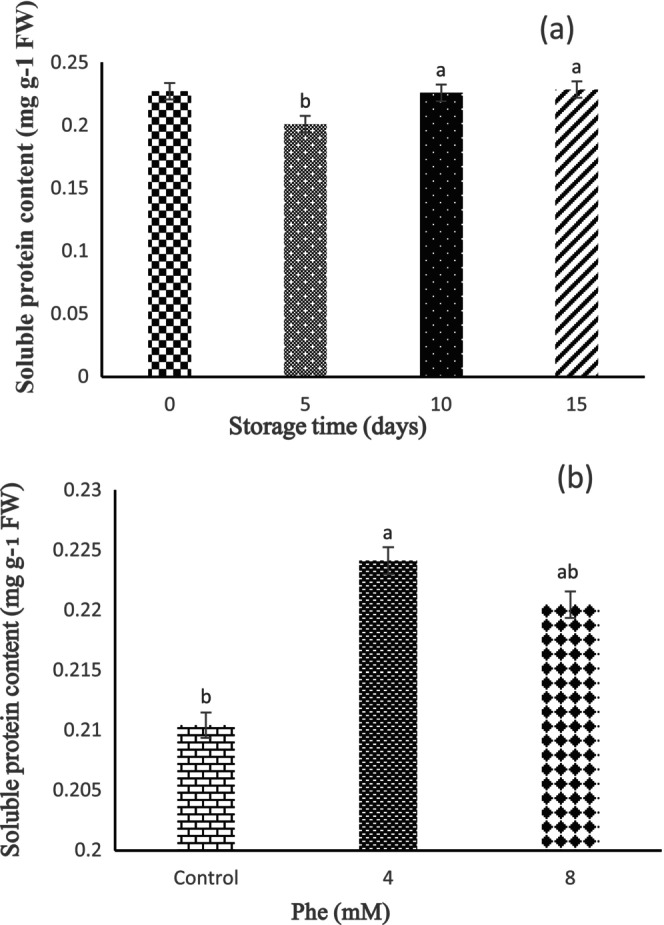
The effect of storage time (a) and L‐phenylalanine (Phe) treatment (b) on soluble protein content of Sabrina strawberry fruits.

Based on the results, the effect of storage time on soluble protein content was statistically significant (*p* < 0.01). According to these results, the lowest soluble protein content was observed in 5 days of storage, and the highest soluble protein content was seen in 15 days of storage (Figure 7a). Overall, an increasing trend was revealed during the storage period. Also, content of soluble protein was statistically significant by Phe treatments (*p* < 0.05). The results revealed that the highest and lowest content of soluble protein was detected in 4 and 8 mM Phe treatments, respectively (Figure 7b).

### 3.5 SOD Enzyme Activity

**FIGURE 8:**
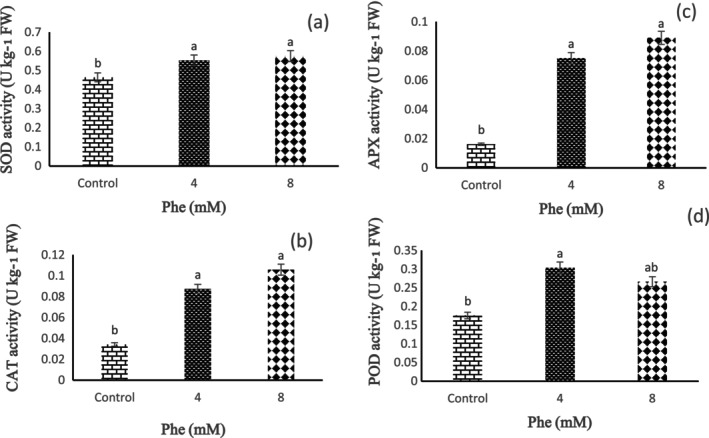
The effect of L‐phenylalanine (Phe) on SOD (a), CAT (b), APX (c), and POD (d) enzymes activity of Sabrina strawberry fruits.

According to the obtained results, we observed that the effect of Phe treatment on SOD enzyme activity was statistically significant (*p* < 0.01). The results showed that strawberry fruits treated with Phe treatments showed an increasing trend in SOD compared to the untreated fruits during storage. Based on our findings, the highest and lowest activity of the SOD enzyme was observed in 8 and 4 mM Phe treatments, respectively (Figure 8a).

### 3.6 CAT Enzyme Activity

Based on our results, it was determined that Phe treatment has a statistically significant effect on CAT enzyme activity (*p* < 0.01). The highest and lowest CAT enzyme activities were revealed in 8 and 4 mM Phe treatments, respectively (Figure 8b). Also, the results showed that strawberry fruits treated with Phe treatments showed an increasing trend in CAT enzyme activity compared to the control during storage.

### 3.7 APX Enzyme Activity

Our results showed a significant effect of Phe treatment on APX enzyme activity (*p* < 0.01). According to these results, the highest activity of the APX enzyme was observed in the treatment of 8 mM Phe, and the lowest activity of the APX enzyme was seen in the treatment of 4 mM Phe. The increasing trend in the activity of APX enzyme in strawberry fruits treated with Phe treatments compared to the control was visible in our results (Figure 8c).

### 3.8 POD Enzyme Activity

Based on the results, Phe treatment has a statistically significant effect on POD enzyme activity (*p* < 0.05). The findings of our study showed that highest and lowest POD activities were observed in 4 and 8 mM Phe treatments, respectively, compared to the control during storage (Figure 8d).

### 3.9 PAL Enzyme Activity

**FIGURE 9:**
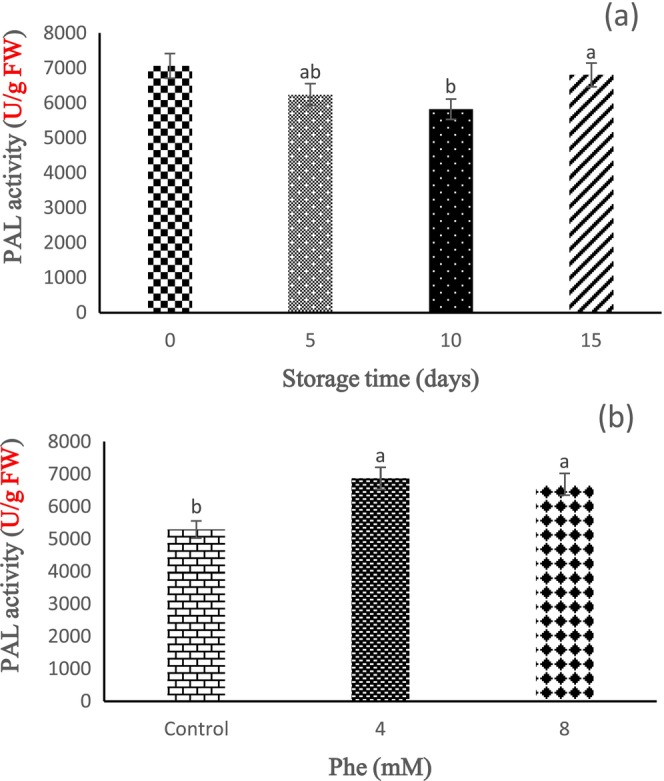
The effect of storage time (a) and L‐phenylalanine (Phe) treatment (b) on PAL enzyme activity of Sabrina strawberry fruits.

Based on our findings, the effect of storage time on PAL enzyme activity was statistically significant (*p* < 0.05). These results showed that PAL enzyme activity decreased during 5 and 10 days of storage compared to the control, but an increasing trend was seen in 15 days of storage (Figure 9a). Also, the effect of Phe treatment on PAL enzyme activity was statistically significant (*p* < 0.05). The results showed that the activity of the PAL enzyme was higher in the fruits treated with Phe compared to the control, and the highest and lowest PAL enzyme activities were observed in the 4 and 8 mM Phe treatments (Figure 9b).

### 3.10 Correlation Analysis

Based on the results (Table 1), WeL showed a positive and highly significant correlation with Pro (*r* = 0.544**). Also, TF with AA (*r* = 0.497**), TP (*r* = 0.604**), TA (*r* = 0.423*), AsA (*r* = 0.595**), PAL (r‐582**), CAT (*r* = 0.550**), APX (*r* = 593**), SOD (*r* = 0.572**), and POD (*r* = 0.407*) had positive and significant correlation. However, TF had a negative correlation with MDA (*r* = −0.559**). Also, the correlation results showed that AA was negatively correlated with MDA (*r* = −0.663**), while with TA (*r* = 0.485*), AsA (*r* = 0.407*), CAT (*r* = 0.392*), APX (*r* = 0.484*), and Pro (*r* = 503**) had a positive and significant correlation. In addition, MDA had a negative and significant correlation with TP (*r* = −0.640**), TA (*r* = −0.498**), AsA (*R* = −0.425*), PAL (*r* = −0.608**), CAT (*r* = −0.471*), APX (*r* = −0.635**), SOD (*r* = −0.462*), and POD (*r* = −0.499**). According to the results, TP had a positive and significant correlation with TA (*r* = 0.550**), AsA (*r* = 0.610**), PAL (*r* = 0.916**), CAT (*r* = 0.582**), APX (*r* = 0.596** *), SOD (*r* = 0.431*), and POD (*r* = 0.458*), whereas TA showed positive and significant correlation with AsA (*r* = 0.569**), PAL (*r* = 0.472*), APX (*r* = 0.463*) and Pro (*r* = 0.542**). Also, AsA had a positive and significant correlation with PAL (*r* = 0.570**), CAT (*r* = 0.465*), APX (*r* = 0.805**), and POD (*r* = 0.541**). PAL was also positively correlated with CAT (*r* = 0.583**), APX (*r* = 0.534**), and POD (*r* = 0.493**). CAT enzyme also showed a positive and significant correlation with APX (*r* = 0.413*) and SOD (*r* = 0.496**), whereas APX had a positive and highly significant correlation with SOD (*r* = 0.523**) and POD (*r* = 0.560**) (Table 1).

**TABLE 1:** Pearson's correlation coefficients between measured traits in strawberry fruits under L‐phenylalanine treatment during storage.

	WeL	TF	AA	MDA	TP	TA	AsA	PAL	CAT	APX	SOD	POD	Pro
WeL	1	0.153	−0.007	0.037	0.129	0.299	0.041	0.059	0.260	−0.108	0.019	−0.121	0.544**
TF		1	0.497**	−0.599**	0.604**	0.423*	0.595**	0.582**	0.550**	0.593**	0.572**	0.407*	0.278
AA			1	−0.663**	0.260	0.485*	0.407*	0.253	0.392*	0.484*	0.362	0.370	0.503**
MDA				1	−0.640**	−0.489**	−0.425*	−0.608**	−0.471*	−0.635**	−0.462*	−0.499**	−0.199
TP					1	0.550**	0.610**	0.916**	0.582**	0.596**	0.431*	0.458*	0.162
TA						1	0.569**	0.472*	0.289	0.463*	0.308	0.265	0.542**
AsA							1	0.570**	0.465*	0.805**	0.298	0.541**	0.321
PAL								1	0.583**	0.534**	0.377	0.493**	0.169
CAT									1	0.413*	0.496**	0.194	0.323
APX										1	0.523**	0.560**	0.187
SOD											1	0.034	0.162
POD												1	0.026
Pro													1

Abbreviations: AA, antioxidant activity; APX, ascorbate peroxidase enzyme; AsA, ascorbic acid; CAT, catalase enzyme; MDA, malondialdehyde content; ns, no significant; PAL, phenylalanine ammonialyase enzyme; POD, guaiacol peroxidase enzyme; Pro, total soluble protein content; SOD, superoxide dismutase enzyme; TA, total anthocyanin content; TF, total flavonoid content; TP, total phenol content; WeL, weight loss.

*Significant at *p* ≤ 0.05; **significant at *p* ≤ 0.001.

### 3.11 Principal Component Analysis

**FIGURE 10:**
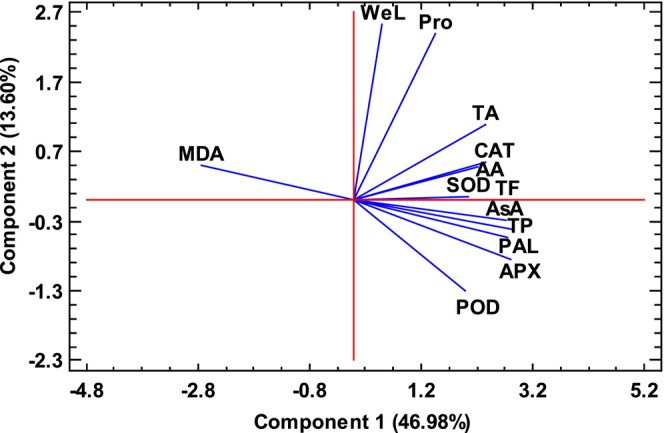
Scores and loading plot for PC1 and PC2 in measured traits of strawberry fruits under L‐phenylalanine treatment during storage. The percentage of total variance explained from each principal component is shown in parentheses. AA, antioxidant activity; APX, ascorbate peroxidase enzyme; AsA, ascorbic acid; CAT, catalase enzyme; MDA, malondialdehyde content; PAL, phenylalanine ammonialyase enzyme; POD, guaiacol peroxidase enzyme; Pro, total soluble protein content; SOD, superoxide dismutase enzyme; TA, total anthocyanin content; TF, total flavonoid content; TP, total phenol content; WeL, weight loss.

Principal component analysis (PCA) was performed here as a statistical tool to evaluate and determine multivariate dependence among the selected variables. Higher factor loading scores mean that there is a tighter association with the same principal component. According to the results, the first two significant PCAs explained 60.58% (46.98% and 13.60%, respectively) of the total variance of the investigated parameters. As shown, PC1 was positively correlated with WeL, Pro, TA, CAT, AA, SOD, TF, AsA, TP, PAL, APX, and POD, whereas PC2 had a positive correlation with MDA. Figure 10 presents the distribution of these data for PC2 and PC2.

## 4 Discussion

Fruit weight is one of the important indicators of fruit quality, which decreases over time due to storage conditions. In the postharvest period, two important factors cause the loss of water and weight loss of the product: first, the interruption of the water connection between the fruit and the mother plant, and second, the increase in transpiration from the surface of the fruit, which is a physiological process and leads to the loss of moisture in the product (Treviño Garza et al. 2015). One of the important features for product quality evaluation is weight loss, which is related to the amount of transpiration and respiration of the fruit. Strawberry fruit is prone to water loss due to its thin skin, which leads to a negative effect on the marketability of the product and causes softening of the texture, change in the color and flavor of the fruit, acceleration of the aging process, growth of pathogens, and frostbite (De Bruno et al. 2023). The loss of fruit weight in the storage time because of the increase in evaporation and transpiration, the non‐uniformity of the water vapor pressure in the intercellular space of the tissues, and the atmosphere surrounding the fruit is a natural process (Quintana et al. 2021; Faz et al. 2016). A previous survey reported that Phe treatments prevent weight loss of blueberry fruit (Ahmadkhani et al. 2023). Similar results were obtained in the present study.

Phe triggers the phenylpropanoid pathway, and then many compounds such as anthocyanins, flavonoids, and dihydrochalcones are produced through the pathway, which maintain plants against various stresses (Vogt 2010) and most of these compounds have high antioxidant properties and are also antifungal (Treutter 2006). Phenol is a simple compound with different properties produced naturally by plants. On the other hand, in response to various stresses, plants produce phenolic compounds as a defense system. Phenolic compounds are known as antioxidants by trapping ROS and have an important effect on fruit quality through changes in the color, aroma, and flavor of fruit products (Al‐Juhaimi, Ghafoor, and Babiker 2012). In addition, phenolic compounds can be included among the most important secondary metabolites in plants, whose biosynthesis is carried out in the skin of fruit and other plant tissues from the two pathways of shikimic acid and acetate‐malonate under the influence of plant growth stage and external factors (Mihan, Sarikhani, and Gholami 2019). Previous studies showed that total phenol (TP) content is positively correlated with antioxidant capacity (Ghasemnezhad, Nezhad, and Gerailoo 2011). In this study, we also observed that TF content positively correlates with antioxidant activity (AA).

Flavonoid compounds obtained through the phenylpropanoid pathway can cause improved defense systems against various stresses (Dixon and Paiva 1995; Patel et al. 2020). Ripening, growth conditions, and genotype can affect the synthesis of phenolic compounds in fruits, and synthesis continues during postharvest storage (Fawbush, Nock, and Watkins 2009). One of the important tissue damages that may occur due to plants being exposed to stress is the increase of free oxygen species. Excessive accumulation of ROS reduces membrane fluidity, ion transport, enzyme activity, and protein synthesis, and leads to cell death (Ranjbar Malidarreh et al. 2019). The reduction of ROS may be carried out by antioxidant compounds such as phenols and flavonoids induced through phenylpropanoid pathway. In general, the increase in capacity and antioxidant activity is directly related to the increase in storage time (Sowmyashree et al. 2021). Antioxidants are compounds that retard or prevent the destruction of primary metabolites including carbohydrates, proteins, lipids, and ribonucleic acid via donating electrons to free radicals (Wang, Luo, and Zhao 2013). As fruits mature, antioxidant levels increase, sometimes as a self‐defense mechanism against ripening. The increase in antioxidant content is mainly due to the increase in lipophilic antioxidant compounds (Naeem et al. 2019). Foliar spraying with Phe increased the total anthocyanins and flavonoids content in grapevine berries (Portu et al. 2015) and preserved the berry color in grapevine (Hattori et al. 2019).

Flavonoid and anthocyanin compounds are responsible for the natural resistance of the fruit, which have antioxidant and antifungal activity and are synthesized from the phenylpropanoid pathway with Phe as a precursor (Kumar Patel et al. 2020). Phe functioned as the primary reaction substrate and underwent catalysis by key enzymes, including PAL resulting in the production of anthocyanins. Another portion of Phe was subjected to catalysis by enzymes within competitive metabolic branches, leading to the formation of colorless flavonoids (Kumar Patel et al. 2020). It can be inferred that exogenous Phe probably increased the quantity of phenol, anthocyanin, and flavonoid reaction substrates and subsequently increased their content. In the previous study on blueberry, it was determined that the increase in TF content may be due to the increase in TA content (Bilbao‐Sainz et al. 2024). In the present study, we also observed that TF content positively correlates with TA content. Also, Phe increased total phenol, anthocyanin, flavonoid, and antioxidant capacity in mango (Patel et al. 2023) and apple fruits (Fanyuk et al. 2022). Aghdam et al. (2019) reported that tomatoes treated with 0.5 mM Phe have higher total phenolic and flavonoid content than the control. Our results were consistent with the results of these studies.

Ascorbic acid (AsA) is a natural antioxidant present in fruits and vegetables that generally decreases after long‐term storage (Asgarian et al. 2022; García‐Pastor et al. 2020). Previous studies have determined that phenolic compounds and ascorbic acid (AsA), as strong antioxidant compounds, enrich the antioxidant properties of fruits and thereby significantly contribute to the removal of free radicals (Li et al. 2018; Wang et al. 2018). The stability of ascorbic acid in fruit has been attributed to the protective effect of phenolic compounds (Miller and Rice‐Evans 1997). Many researchers believe AsA content decreases during storage time due to oxidation and fruit respiration rate (De Bruno et al. 2023). The reduction of AsA content in cold storage is related to activity of the ascorbic acid oxidase enzyme, increasing pH, temperature, and oxygen levels (Poraziz et al. 2019). However, Tavarini et al. (2008) showed that ascorbic acid content in fruit after storage could be higher than that in fruit at harvest, which was closely related to harvest time. Also, Wang et al. (2023) concluded that Phe treatment maintained the level of ascorbic acid in pear and increased shelf life. Also, ascorbic acid content is maintained in plum fruit treated with Phe (Sogvar et al. 2020). In accordance with these studies, we revealed Phe treatment maintained AsA content of strawberry fruits during storage time.

MDA is one of the intermediary products in lipid peroxidation and causes damage to the membrane and organelles of fruit cells. Membrane lipid peroxidation, which is related to aging, catalyzes the hydroperoxidation of unsaturated fatty acids by using lipid oxidizing enzymes and converting and decomposing them into reactive oxygen species (ROS) (Wang et al. 2015). Cell membrane integrity can protect plants in defiance of various stresses. Therefore, any damage to the cell membrane through lipid peroxidation by the reactive oxygen radicals (oxidative stress) can be identified through the amount of ion exchange and MDA content as an obvious feature of the response to stress conditions (Gohari et al. 2020). The reduction of MDA indicates greater steadiness and wholeness of cell membranes, which increase the storage life of fruits by mitigating the peroxidation of lipids and supporting membrane integrity and cellular structure (Arefnia, Hatamzadeh, and Ghasemnejad 2019). Aghdam et al. (2019) reported that postharvest treatment of tomato fruits with Phe leads to reduction of cold stress injuries, lipid peroxidation, and accumulation of free radicals. Patel et al. (2023) reported that Phe treatment decreased MDA content, electrolyte leakage, and H_2_O_2_ in mango fruits. Also, Nasr et al. (2021) observed that the Phe treatment decreased MDA and H_2_O_2_ content in persimmon fruits. In agreement with these studies, we also observed that Phe treatment reduces the MDA content of strawberry fruits during storage time.

Protein breakdown is a sign of cell membrane destruction. The increase of oxygen free radicals during aging destroys proteins. AsA as an antioxidant can delay the degradation of proteins by neutralizing ROS (Noctor and Foyer 1998). l‐phenylalanine may slow the rate of degradation by facilitating the synthesis of antioxidant compounds such as anthocyanins. Salim et al. (2021) reported that amino acid treatment increased soluble protein content in tomato plant leaves and increased yield. We also concluded that Phe treatment increases the content of soluble protein, as a result, the shelf life of treated strawberry fruits is improved by reducing the damage to the cell membrane.

Various mechanisms to deal with ROS are found in living organisms, one of the most important is the enzymatic antioxidant system includes catalase (CAT), guaiacol peroxidase (POD), and superoxide dismutase (SOD). CAT is an enzyme found in all living organisms, including plant and animal cells and aerobic microorganisms. Removal of excess amounts of H_2_O_2_ and interference in regulating the capacity of appropriate cellular H_2_O_2_ is the responsibility of two enzymes, CAT and POD, the CAT enzyme plays an effective role in this function. CAT catalyzes the conversion of H_2_O_2_ into water and oxygen (Van Assche and Clijsters 1990; Prassad 1997). Also, APX as an antioxidant participates in the glutathione ascorbate cycle, which, like SOD and CAT enzymes, plays a vital role in reduction of ROS (Hasanuzzaman and Fujita 2013). SOD provides a frontline defense against ROS by scavenging O_2_ (Hasanuzzaman et al. 2012). CAT, SOD, and APX enzymes as antioxidant can eliminate excess free radicals and reduce cellular destruction induced via oxidative stress (Tang et al. 2022). SOD enzyme is responsible for destroying the superoxide radicals in cells under stress and converting them into oxygen and hydrogen peroxide. Then hydrogen peroxide is converted into water and oxygen by CAT. APX also decomposes hydrogen peroxide by receiving electrons from ascorbic acid through the ascorbic acid/glutathione cycle (Pan et al. 2019). PODs are present in cytosol, vacuole, chloroplast and apoplast and have an important and special role in the antioxidant defense system (Poddar et al. 2018). Previously, Sogvar et al. (2020) revealed that plums treated with 7.5 mM Phe indicated higher SOD, APX, and CAT activities and lower hydrogen peroxide content. Xie et al. (2022) detected that Phe treatment elevated the activity of POD in melon. In another study, Phe elevated the activities of CAT, APX, and GR in mango fruits more than the control (Patel et al. 2023). Also, Aghdam et al. (2019) stated that tomato fruits treated with Phe showed that antioxidant enzymes such as CAT, GR, APX, and SOD increased compared to untreated fruits. In agreement with these studies, we also observed that Phe treatment increased the activities of CAT, POD, APX, and SOD enzymes in stored strawberry fruits.

Phenolic compounds as a natural antioxidant are produced directly through the phenylpropanoid biosynthesis pathway (Asghari 2019). L‐phenylalanine, as an exogenous factor, can directly regulate the gene expression patterns of key enzymes in metabolic pathways. The PAL enzyme is one of the main enzymes in synthesizing phenolic compounds that activate the phenylpropanoid pathway (Asghari and Zahedipour 2016). The first compound produced through the phenylpropanoid pathway is transcinnamic acid, which is obtained from the amino acid Phe by the PAL enzyme, following many secondary metabolites such as phenol, lignin, phytoalexins, and flavonoids are also produced (Bagal et al. 2012). Phenolic compounds biosynthesis starts from the aromatic amino acid called Phe, which is carried out by deamination of Phe by the PAL and its conversion to transcinnamic acid (Michalak 2006). Therefore, treating fruits with Phe strengthens the phenylpropanoid pathway. Sogvar et al. (2020) revealed that Phe treatment increased PAL enzyme activity in plum fruits. Also, PAL enzyme activity increased in tomato fruits treated with Phe (Aghdam et al. 2019). Our results were also consistent with the results of these studies. Overall, our findings showed that Phe treatment increases defense systems and antioxidant compounds and can be used as a suitable treatment to increase the postharvest life of strawberry fruits. Furthermore, according to the PCA results, it was found that phenolic and antioxidant compounds increase the quality and shelf life of strawberry fruit by reducing MDA levels. On the other hand, it can be observed that there is a negative relationship between these variables and MDA content.

## 5 Conclusions

This research investigated the biochemical and antioxidant compounds of strawberry fruits treated with Phe during cold storage. Our finding revealed Phe reduced the accumulation of MDA and prevented fruit weight loss. The process of fruit aging was slowed down by maintaining and increasing the content of soluble protein, antioxidant capacity, TP, TF, TA content, and ascorbic acid. As a result, postharvest life and antioxidant activities of strawberry fruits treated with Phe were improved by increasing PAL, CAT, SOD, APX, and POD activities. Therefore, Phe treatment maintains strawberry fruit quality and improves postharvest life. Based on these results, further research about identifying various phytochemicals such as phenolic, amino acids, and fatty acid profiles in strawberry fruits under L‐phenylalanine treatment during storage and determining their biological activity should be carried out in the future.

## Author Contributions

**Karim Manda‐Hakki:** investigation (equal), visualization (equal), writing – original draft (equal). **Hamid Hassanpour:** conceptualization (equal), data curation (equal), methodology (equal), supervision (equal), validation (equal), writing – review and editing (equal).

## Conflicts of Interest

The authors declare no conflicts of interest.

## Data Availability

The datasets used and/or analyzed during the current study are available from the corresponding author upon reasonable request.
